# Carotid Flow Time Analysis as a Method to Predict Fluid Responsiveness in Mechanically Ventilated Children

**DOI:** 10.1111/pan.15136

**Published:** 2025-06-02

**Authors:** Humberto M. Silva, Raisa S. Uzun, Victoria C. Lintz, Roberto J. N. Nogueira, Tiago H. De Souza

**Affiliations:** ^1^ Pediatric Intensive Care Unit, Department of Pediatrics State University of Campinas (UNICAMP) Campinas SP Brazil; ^2^ Department of Internal Medicine School of Medical Sciences of the State University of Campinas (UNICAMP) Campinas SP Brazil

**Abbreviations**
AUROC: area under the receiver operating characteristic curve
BSA: body surface area
CFTc: corrected carotid flow time
CFTc‐B: corrected carotid flow time using Bazett's formula
CFTc‐W: corrected carotid flow time using Wodey's formula
CI: confidence interval
HR: heart rate
IQR: interquartile range
mL/kg: milliliters per kilogram
ms: milliseconds
NLR: negative likelihood ratio
NPV: negative predictive value
OR: Odds Ratio
PICU: pediatric intensive care unit
PLR: positive likelihood ratio
PPV: positive predictive value
ROC: receiver operating characteristic
TTE: transthoracic echocardiography
UNICAMP: Universidade Estadual de Campinas (State University of Campinas, Brazil)

Recently, the variation in peak aortic blood flow velocity has been recognized as the most accurate ultrasonographic method for assessing fluid responsiveness in children [1]. However, it is technically complex and requires advanced training in point‐of‐care ultrasound. Additionally, it may be unreliable in up to 30% of cases due to poor acoustic windows, often a consequence of invasive mechanical ventilation [2]. To address the limitations of transthoracic echocardiography (TTE), Doppler ultrasound of the common carotid artery has been proposed as an accurate alternative for predicting fluid responsiveness in mechanically ventilated children.

This secondary analysis used data from a prospective cohort study conducted at the Pediatric Intensive Care Unit (PICU) of the Clinics Hospital, State University of Campinas (UNICAMP), Brazil. The study was approved by the ethics committee (approval #12894719.8.0000.5404), and informed consent was obtained.

For full methodological details, refer to the original study and Appendix S1 [3]. Briefly, mechanically ventilated children with a tidal volume of 8–10 mL/kg requiring fluid bolus administration were included. The decision to administer fluids was at the attending physician's discretion, based on clinical signs of hypoperfusion. TTE and left carotid artery Doppler ultrasound were performed before and after a 10 mL/kg crystalloid infusion over 10 min. Stroke volume and corrected carotid flow time (CFTc) were measured in triplicate, with the average used for analysis. Patients were classified as fluid responders if stroke volume increased by ≥ 15% after the bolus. The CFTc was calculated using Bazett's formula (CFTc‐B = $\text{flow time}/\sqrt{\text{cycle time}}$) and Wodey et al.'s formula (CFTc‐W = flow time + 1.29 × [HR − 60]).

All 30 participants from the primary study were included in this secondary analysis. The median age was 20 months (IQR 7.0–65.0) and the median weight was 10 kg (IQR 6.0–20.0). An increase in stroke volume greater than 15% following volume expansion was observed in 12 patients (responders).

Before volume expansion, median CFTc‐B was lower in responders than in nonresponders [290 ms (IQR 254–317) vs. 329 ms (IQR 307–343); *p* = 0.038]. In contrast, CFTc‐W showed no significant difference between responders and nonresponders [300 ms (IQR 292–332) vs. 318 ms (IQR 306–339); *p* = 0.086]. ROC curve analysis identified CFTc‐B, CFTc‐B adjusted for body surface area (CFTc‐B/BSA), and CFTc‐W/BSA as predictors of fluid responsiveness (Figure 1). A detailed analysis of predictor performance is presented in Table 1.

**FIGURE 1:**
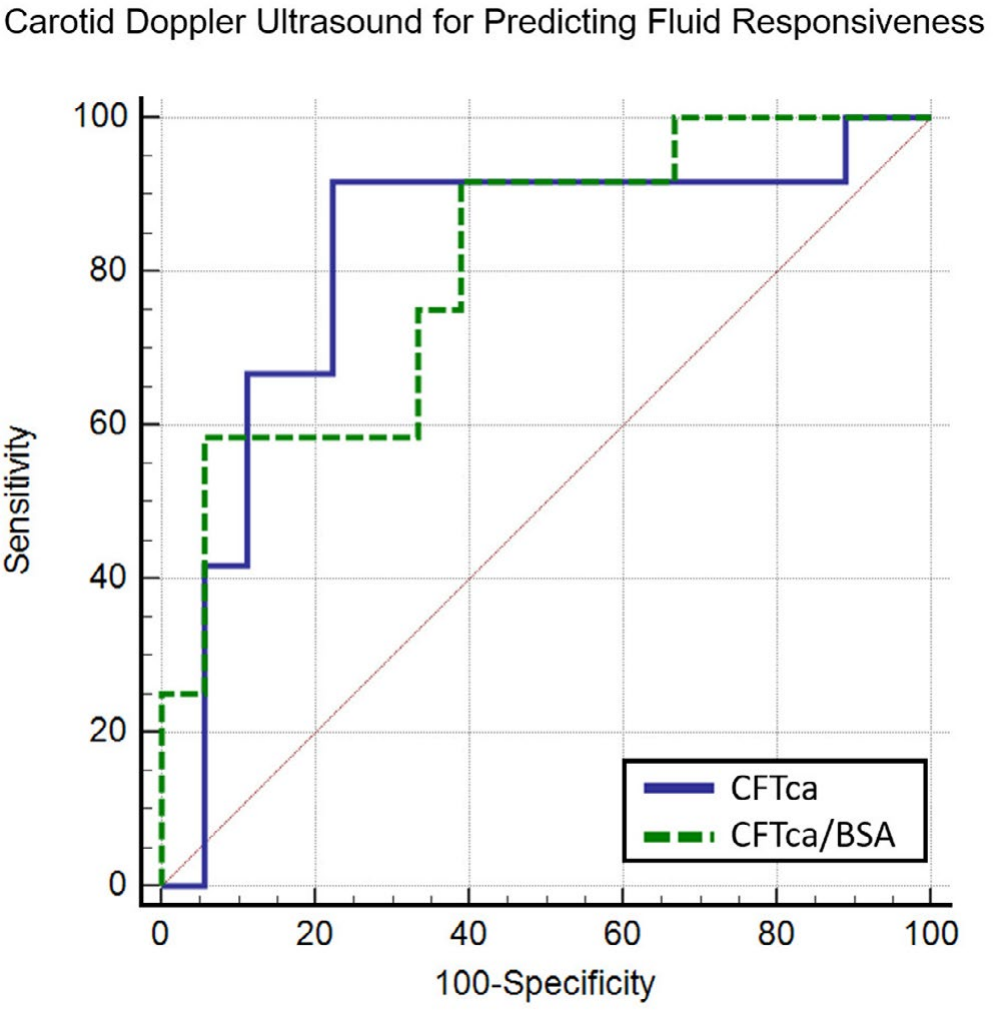
ROC curve analysis of variables obtained from carotid Doppler ultrasound for predicting fluid responsiveness.

**TABLE 1:** Areas under the ROC curve for assessed predictors of fluid responsiveness. Cut point calculated using Youden's index. Values are expressed as median (25th–75th percentiles).

Performance	CFTc‐B	CFTc‐B/BSA	CFTc‐W/BSA
AUROC (95% CI)	0.82 (0.64–0.93)	0.81 (0.62–0.93)	0.77 (0.58–0.90)
Optimal cutoff	312 ms	335.8 ms m^−2^	364.0 ms m^−2^
Sensitivity (%)	91.7%	58.3%	50.0%
Specificity (%)	77.8%	94.4%	94.4%
PPV	73.3%	87.5%	85.7%
NPV	93.3%	77.3%	73.9%

Abbreviations: AUROC, area under the receiver operating characteristic curve; BSA, body surface area; CFTc‐B, corrected carotid blood flow using Bazett's formula; CFTc‐W, corrected carotid blood flow using Wodey's formula; CI, confidence interval; NLR, negative likelihood ratio; NPV, negative predictive value; PLR, positive likelihood ratio; PPV, positive predictive value.

In the univariate analysis, cardiac index, stroke volume index, weight, CFTc‐B, CFTc‐B/BSA, and CFTc‐W/BSA were all significantly associated with fluid responsiveness (*p* < 0.05). After evaluating multicollinearity, cardiac index, weight, CFTc‐B, and CFTc‐B/BSA were included in a multivariate logistic regression model. In the stepwise regression, only CFTc‐B/BSA was retained (OR = 0.99; 95% CI 0.993–0.999; *p* = 0.002). Therefore, each 1‐ms increase in CFTc‐B/BSA is associated with a 1% reduction in the likelihood of fluid responsiveness. The group of patients with CFTc‐B/BSA ≤ 335.8 ms had increased fluid responsiveness, with an odds ratio of 11.2 (95% CI, 1.73–72.3).

In this study, we found that CFTc measured via Doppler ultrasound shows promise as a reliable method for predicting fluid responsiveness in mechanically ventilated children. To our knowledge, only one previous study has assessed the accuracy of CFTc as a predictor of fluid responsiveness in children [4]. In 2022, Lin et al. studied children under general anesthesia receiving positive pressure ventilation (PEEP 10 cmH_2_O, tidal volume 8 mL/kg) who were given a 10 mL/kg crystalloid bolus. They observed that CFTc increased in both responders and nonresponders, with no correlation between CFTc changes and fluid responsiveness. These findings may have been influenced by their definition of fluid responsiveness. While most pediatric studies using TTE define it as a ≥ 15% increase in stroke volume, Lin et al. used a 10% threshold, which may not be sufficient for accurate detection [1, 3].

While promising, this study has limitations. First, TTE was used as the reference instead of gold‐standard methods like Fick or thermodilution. However, pediatric Doppler‐based cardiac output measurements have shown acceptable accuracy, precision, and repeatability [5]. Second, while carotid Doppler ultrasound is less affected by respiratory movements, technical artifacts may still introduce bias. Third, the study included a wide age range, with some patients on vasoactive medications, highlighting the need for studies in more homogeneous groups. Fourth, as it focused on sedated, mechanically ventilated patients, the findings may not apply to those breathing spontaneously. Lastly, like all ultrasound methods, accuracy depends on the operator. Although a single operator conducted all measurements to minimize bias, further research is needed to evaluate intra‐ and inter‐operator reliability.

In conclusion, our findings indicate that CFTc has significant potential as a predictor of fluid responsiveness in pediatric patients on mechanical ventilation, offering a viable alternative when conventional methods like TTE are not feasible.

## Author Contributions

Humberto M. Silva, Raisa S. Uzun, and Victoria C. Lintz: responsible for data collection, drafting, and critical revision of the manuscript. Roberto J.N. Nogueira: responsible for critical revision of the manuscript for important intellectual content. Tiago H. De Souza: responsible for the study concept and design, acquisition, analysis, and interpretation of data.

## Disclosure

Consent to participate: Written informed consent was obtained from the participants' legal guardian.

## Ethics Statement

The study was approved by the local institutional review board (UNICAMP's Research and Ethics Committee, approval number 12894719.8.0000.5404).

## Consent

The authors have nothing to report.

## Conflicts of Interest

The authors declare no conflicts of interest.

## Supporting information


Appendix S1

## Data Availability

The data that support the findings of this study are available from the corresponding author, upon reasonable request.

## References

[1] F. L. Carioca , F. M. de Souza , T. B. de Souza , et al., “Point‐Of‐Care Ultrasonography to Predict Fluid Responsiveness in Children: A Systematic Review and Meta‐Analysis,” Pediatric Anesthesia 33, no. 1 (2023): 24–37. 36222022 10.1111/pan.14574

[2] J. R. Egan , M. Festa , A. D. Cole , G. R. Nunn , J. Gillis , and D. S. Winlaw , “Clinical Assessment of Cardiac Performance in Infants and Children Following Cardiac Surgery,” Intensive Care Medicine 31, no. 4 (2005): 568–573. 15711976 10.1007/s00134-005-2569-5

[3] T. B. de Souza , A. J. Rubio , F. L. Carioca , et al., “Carotid Doppler Ultrasonography as a Method to Predict Fluid Responsiveness in Mechanically Ventilated Children,” Pediatric Anesthesia 32, no. 9 (2022): 1038–1046. 35748620 10.1111/pan.14513

[4] E. E. Lin , C. Glau , T. W. Conlon , et al., “The Association Between Carotid Flow Time and Fluid Responsiveness in Children Under General Anesthesia,” Pediatric Anesthesia 32, no. 9 (2022): 1047–1053. 35735131 10.1111/pan.14510

[5] M. S. Chew and J. Poelaert , “Accuracy and Repeatability of Pediatric Cardiac Output Measurement Using Doppler: 20‐Year Review of the Literature,” Intensive Care Medicine 29, no. 11 (2003): 1889–1894. 12955181 10.1007/s00134-003-1967-9

