# Supplementary material for: Carotid Flow Time Analysis as a Method to Predict Fluid Responsiveness in Mechanically Ventilated Children
Source: Paediatr Anaesth. 2025 Jun 2;35(9):787–9. doi: 10.1111/pan.15136 (PMC12340333; doi:10.1111/pan.15136)
Supplement: Supplementary file 1 — Appendix S1 [file PAN-35-787-s001.docx]

***Carotid flow time analysis as a method to predict fluid responsiveness in mechanically ventilated children***

Humberto M. Silva, MD^a^; Raisa S. Uzun, MD^a^; Victoria C. Lintz, MD^a^; Isabel de Siqueira Ferraz, MD, MSc^a^; Roberto J. N. Nogueira, MD, PhD^b^ and Tiago H. de Souza, MD, PhD^a^

***Affiliations:***

^a^ Pediatric Intensive Care Unit, Department of Pediatrics, State University of Campinas (UNICAMP), Campinas, SP, Brazil;

^b^ Department of Internal Medicine, School of Medical Sciences of the State University of Campinas (UNICAMP), Campinas, SP, Brazil.

**METHODS**

***Study design, subjects, and setting***

This secondary analysis utilizes data from a prospective cohort study conducted at the Pediatric Intensive Care Unit (PICU) of the Clinics Hospital, State University of Campinas (UNICAMP), a quaternary care academic hospital located in São Paulo, southeastern Brazil. The study was approved by the UNICAMP Research Ethics Committee (approval #12894719.8.0000.5404), and written informed consent was obtained from the legal guardians of all participants.

Participants in this secondary analysis were selected according to the eligibility criteria defined in the primary study. All mechanically ventilated children requiring fluid administration were screened consecutively. The decision to provide fluids was made by the attending physician as part of routine care, based on clinical indications of insufficient tissue perfusion, including tachycardia, hypotension, oliguria, delayed capillary refill, or hemodynamic instability despite the use of vasoactive agents.

The inclusion criteria for the primary study were: 1) tidal volume between 8-10 mL/kg; 2) positive end-expiratory pressure of 5-6 cmH_2_O; 3) absence of spontaneous breathing; and 4) sinus rhythm. Exclusion criteria included: 1) congenital heart defects; 2) left ventricular systolic dysfunction (ejection fraction < 50%); 3) anatomical abnormalities of the neck; 4) contraindications for cervical mobilization (e.g., post-surgical care following head or neck surgery, traumatic brain injury, or spinal cord injury); 5) suspected intracranial hypertension; 6) skin lesions or bandages at ultrasound or echocardiography examination sites; 7) poor echocardiographic windows; and 8) unavailability of an operator or ultrasound equipment during participant enrollment.

***Study protocol***

Transthoracic echocardiography (TTE) and Doppler ultrasound of the left carotid artery were performed immediately before and after the infusion of 10 mL/kg of crystalloid solution (either normal saline or lactated Ringer's solution) over a 10-minute period. At each data collection point, measurements of stroke volume and corrected carotid flow time were taken in triplicate, with the average value used for analysis. Stroke volumes were measured using transthoracic echocardiography, and patients were classified as fluid responders if they exhibited at least a 15% increase in stroke volume after the fluid bolus. This 15% threshold was selected based on previous studies^1^.

***Ultrasound measurements***

Ultrasound examinations, including transthoracic echocardiography (TTE) and carotid Doppler ultrasound (CDU), were conducted using a Vivid Q scanner (GE Healthcare, Tirat Carmel, Israel). The TTE utilized a phased array transducer (3.5–8 MHz), while the CDU employed a linear transducer (5–13 MHz). Although both procedures were performed simultaneously, the analysis of the relevant variables was conducted later, ensuring that the operator was blinded to the TTE and CDU results during data collection. All ultrasound exams were carried out by a skilled operator, who also served as an instructor for the pediatric point-of-care ultrasound (POCUS) course offered by the Brazilian Society of Intensive Care.

B-mode images of the left common carotid artery (CCA) were acquired in the long-axis view at the level of the thyroid gland. Spectral Doppler tracings were obtained by positioning a 0.5 mm sample volume at the center of the vessel. Although corrected carotid flow time is independent of insonation angle, angle correction was aligned parallel to the CCA wall, with a maximum insonation angle of 60º.

The aortic velocity-time integral (VTI) was measured from an apical five-chamber view using pulsed Doppler at the same level. The VTI was determined by automatically tracing Doppler waveforms during a single respiratory cycle, with the average of the highest and lowest values being recorded. To calculate left ventricular stroke volume (SV), the mean of three VTI measurements was used. The following formula was applied: $SV (ml)= \left( \pi x {Da}^{2}/4 \right) x VTI$, where Da and VTI are in centimeters. Cardiac index (Ci) was then calculated as $Ci= \frac{SV x heart rate}{body surface area}$.

The corrected carotid flow time (CFTc) was calculated by measuring the systolic flow time through the carotid artery, typically in milliseconds, and adjusting it for heart rate using Bazett's formula. In this formula, the carotid flow time (CFTc-B) was expressed in milliseconds (ms), and the RR interval, which is the time between two consecutive R-waves on the ECG, was expressed in seconds (s): $CFTc-B= \frac{Carotid Flow Time (ms)}{\sqrt{RR interval (s)}}.$ Additionally, CFTc was calculated using Wodey et al.'s formula: $CFTc-W=Carotid Flow Time \left( ms \right)+1.29 x \left( heart rate-60 \right).$

***Statistical Analysis***

Statistical analysis and sample size calculation was performed using MedCalc Statistical Software version 19.8 (MedCalc Software bvba, Ostend, Belgium). Parte inferior do formulário

Data normality was assessed using the Kolmogorov–Smirnov and Shapiro-Wilk tests. Continuous variables were reported as medians with interquartile ranges (IQR), while categorical variables were presented as absolute numbers and percentages. Pre- and post-volume expansion comparisons of continuous variables were made using the Wilcoxon rank-sum test, and differences between responders and non-responders were evaluated with the Mann–Whitney U-test. Categorical variables were analyzed using the chi-square or Fisher's exact test. ROC curves were generated for the fluid responsiveness predictors and areas under the ROC curves (AUROC) were calculated and compared using the non-parametric method proposed by DeLong et al.^2^. Youden’s J statistic was used to determine optimal cut-off points. Relationships between the predictors and changes in SV induced by fluid infusion were assessed using Spearman's correlation coefficient (ρ). A multivariate logistic regression analysis using the stepwise method was conducted to identify variables associated with the primary outcome by estimating odds ratios (OR) and 95% confidence intervals (CI). Variables that showed a significant association with the primary outcome in the univariate analysis (*p*<0.05) were included in the initial model. Statistical significance was accepted for *p*-values of <0.05.

This study represents a secondary analysis of data collected in a previously published study. Therefore, no new sample size calculation was performed for this analysis. In the original study, the sample size calculation was based on a fluid responsiveness rate of 33%, as determined by previous pediatric studies. Considering an α error of 0.05 and a statistical power of 80%, 30 participants were needed to detect a 0.30 difference between the area under the receiver operating characteristic curve (AUROC) of respiratory variation in carotid blood flow peak velocity and the null hypothesis (AUROC = 0.5; i.e., no discriminative power). A post-hoc power analysis was conducted to evaluate the statistical power of the primary analysis, which focused on the AUROC of CFTc for predicting fluid responsiveness. The analysis used the observed AUROC and maintained the same significance level (α = 0.05) as the original study. The post-hoc analysis indicated a power of 85%, reflecting the adequacy of the sample size for this primary outcome.

**REFERENCES**

1 Carioca F de L, de Souza FM, de Souza TB, Rubio AJ, Brandão MB, Nogueira RJN, et al. Point-of-care ultrasonography to predict fluid responsiveness in children: A systematic review and meta-analysis. Pediatric Anesthesia. John Wiley & Sons, Ltd; 2023 Jan 1;33(1):24–37.

2 DeLong ER, DeLong DM, Clarke-Pearson DL. Comparing the areas under two or more correlated receiver operating characteristic curves: a nonparametric approach. Biometrics. 1988 Sep;44(3):837–45.


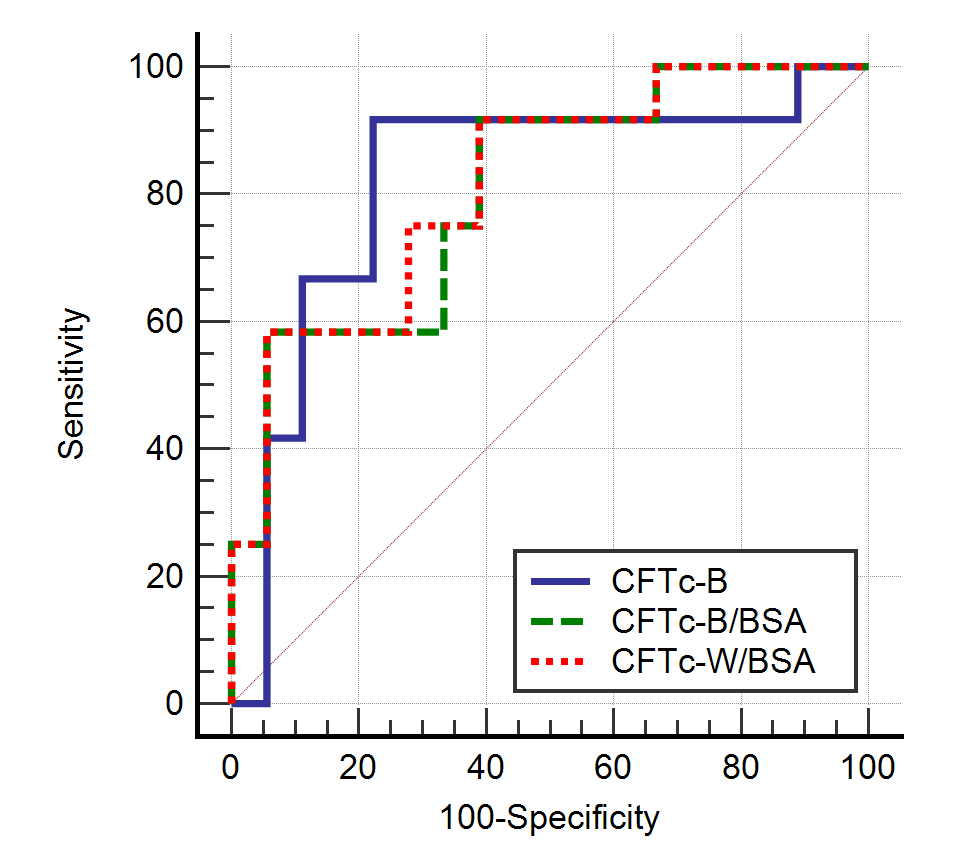


Figure 1 – Receiver operating characteristic (ROC) curve analysis results showing the area under the curve (AUROC) for CFTc-B (0.82, 95% CI: 0.64–0.93), CFTc-B/BSA (0.81, 95% CI: 0.62–0.93), and CFTc-W/BSA (0.77, 95% CI: 0.58–0.90).
